# Explorative study on *Lactobacillus* species and their acid‐producing capacity and anti‐microbial activity in head and neck cancer patients

**DOI:** 10.1002/cre2.426

**Published:** 2021-03-31

**Authors:** Matilda Rahne, Amina Basic, Annica Almståhl

**Affiliations:** ^1^ Department of Oral Microbiology and Immunology, Institute of Odontology, Sahlgrenska Academy University of Gothenburg Gothenburg Sweden

**Keywords:** acid‐producing capacity, anti‐microbial activity, head and neck cancer, *Lactobacillus* species, plaque, tongue

## Abstract

**Objective:**

To determine acid‐producing capacity and anti‐microbial activity of *Lactobacillus* species collected pretreatment and post treatment in head and neck cancer patients.

**Material and Methods:**

*Lactobacillus* isolates from 21 patients pretreatment and post treatment were identified using molecular methods. The patients' stimulated salivary secretion was determined pretreatment, and 6 and 12 months post treatment and caries lesions/new filled surfaces registered at 24 months post treatment. The acid‐producing capacity of the *Lactobacillus* isolate was analyzed using a colorimetric fermentation test in microtiter plates. The anti‐microbial activity of the isolates against *Streptococcus mutans* associated with caries, and against the mucosal pathogens *Staphylococcus aureus*, *Candida albicans*, and *Enterococcus faecalis* was analyzed by determining inhibitory zones on agar plates.

**Results:**

The most frequent species were *L. paracasei* (*n* = 21), *L. casei/rhamnosus* (*n* = 17) and *L. fermentum* (*n* = 10). Sixty‐seven percent of the patients harbored *L. paracasei* either at 6 or 12 months post radiotherapy. The corresponding figures for *L. casei/rhamnosus* and *L. fermentum* were 62% and 33%. *L. paracasei* strains showed the best acid‐producing capacity and *L. fermentum* strains the lowest. Strong acid‐producing capacity was most common among isolates collected at 6 months post treatment. Seventy‐two percent of the strains showed an anti‐microbial activity against *S. mutans*, one strain against *S. aureus* and none against *C. albicans* or *E. faecalis*.

**Conclusion:**

The most frequent species isolated from head and neck cancer patients both pretreatment and post treatment were *L. paracasei*, *L. casei/rhamnosus*, and *L. fermentum. L. paracasei* showed the best acid‐producing capacity and the highest proportion with anti‐microbial activity against *S. mutans*.

## 1 INTRODUCTION

Patients who have undergone treatment for cancer in the head and neck region have an increased risk of especially dental caries (Sroussi et al., 2017). A plausible explanation for this is the reduced salivary secretion rate, which is a consequence of damages to the salivary glands due to radiotherapy (Burlage et al., 2001). A reduced salivary secretion rate might lead to long periods of low pH (Hase & Birkhed, 1988) favoring acidogenic and acid‐tolerant microorganisms. High levels of both mutans streptococci and *Lactobacillus* have been found in irradiated subjects and especially in those with very low stimulated salivary secretion rates (Almståhl et al., 2008).

In most previous studies, only the total number of *Lactobacillus* has been reported and no identification to species level has been performed. In subjects with hyposalivation due to either primary Sjögren's syndrome or to radiotherapy (3–5 years post treatment) the most prevalent species reported were *L. fermentum*, *L. casei*, and *L. rhamnosus* (Almståhl et al., 2010). In subjects with normal salivary secretion rate and good oral health, *L. plantarum*, *L. rhamnosus*, *L. salivarius*, *L. acidophilus*, and *L. gasseri* are frequently found species (Ahumada et al., 2003; Colloca et al., 2000; Köll‐Klais et al., 2005). Most of these species have been found also in subjects with caries; *L. fermentum*, *L. plantarum*, *L. rhamnosus*, *L. casei*, *L. delbrueckii*, *L. salivarius*, *L. vaginalis*, and *L. brevis* (Ahumada et al., 2003; Caufield et al., 2007; Smith et al., 2001).

The ability to produce acids is a common trait among *Lactobacillus* spp. (Piwat et al., 2010). There are, however, variations between different species (Almståhl et al., 2013; Piwat et al., 2010) and also between strains of the same species.

The role of the increased number of *Lactobacillus* species found in the oral cavity of hyposalivated subjects is not fully understood. On the one hand, *Lactobacillus* contribute to an acidic environment, which is undesirable for microorganisms associated with good oral health like streptococci and *Neisseria*. On the other hand, *Lactobacillus* spp. can have anti‐microbial effects. The antimicrobial activity of *L. casei rhamnosus* against several pathogens such as for example *Escherichia coli*, *Enterobacter cloacae*, and *Salmonella typhimurium* has been reported (Forestier et al., 2001). Furthermore, a study on the bacteriocin production of *Lactobacillus* species isolated from plaque and saliva of patients with caries showed that all species except *L. jensenii* produced bacteriocins against at least one bacteria among *E. coli*, *Salmonella* spp, *Klebsiella*, *Shigella dysenteriae*, *S. sonnei*, and *Campylobacter* (Smith et al., 2001). Other studies have also shown that *Lactobacillus* (strains of *L. casei*, *L. fermentum*, *L. plantarum*, *L. paracasei*, *L. salivarius*, *L. rhamnosus*) can have anti‐microbial effects on *Streptococcus mutans* (Ahola et al., 2002; Keller et al., 2011; Köll et al., 2008; Köll‐Klais et al., 2005; Näse et al., 2001). The majority of *Lactobacillus* strains isolated from saliva and subgingivally from healthy subjects were able to suppress the growth of the periodontal pathogens *Aggregatibacter actinomycetemcomitans*, *Porphyromonas gingivalis*, and *Prevotella intermedia* (Köll et al., 2008). In addition, ingestion of cheese containing several probiotic bacteria of which two were *Lactobacillus* strains (*L. rhamnosus* GG and LC705) reduced high levels of *Candida* in elderly subjects (Hatakka et al., 2007).

Studies about the prevalence of different *Lactobacillus* species in the oral cavity before, and after treatment of cancer of the head and neck region are lacking and little is known about their acid‐producing capacity and anti‐microbial activity. Such knowledge is valuable to understand the role of elevated numbers of *Lactobacillus* species in the oral cavity, which in turn might be used in caries preventive strategies for patients who have undergone treatment for cancer in the head and neck region. We hypothesize that *Lactobacillus* isolates collected post treatment in patients with cancer of the head and neck show greater species diversity, have higher acid‐producing capacities, and an increased anti‐microbial effect compared with isolates collected pretreatment.

The aim of this study was to determine the acid‐producing capacity and anti‐microbial activity of *Lactobacillus* species collected pretreatment and post treatment in head and neck cancer patients.

## 2 MATERIAL AND METHODS

In the project entitled “Longitudinal changes in saliva, microflora, diet and oral status in relation to the quality of life in people receiving radiotherapy to the head and neck region” (approved by the Ethical Committee at the University of Gothenburg [Dnr 682–07]), microbial samples were collected from 33 head and neck cancer patients (23 men, 10 women, mean age 59 ± 8 years and the mean number of teeth 25 ± 5 teeth). Microbial samples were collected at pretreatment, and at 6 and 12 months post radiotherapy (RT) by a dentist at the Department of Oral and Maxillofacial Surgery, Gothenburg. Among those 33 patients, 21 patients showed at least moderate growth (described below) of *Lactobacillus* at least at two of the three time‐points (before, 6 months post RT, 12 months post RT) and they were therefore selected for the present study.

### 2.1 Clinical examination

Before starting radiation therapy, manifest caries lesions were restored. Two years post treatment, caries lesions/new filled surfaces were registered using dental records and dental radiographs.

### 2.2 Stimulated salivary secretion rate

The stimulated whole salivary secretion rate was determined using paraffin wax (about 1 cm^3^, melting point 42°C–44°C, Orion Diagnostics). The patient was instructed to chew on a piece of paraffin until it was soft and to swallow once. Thereafter, the patient chewed on the paraffin wax at his/her own pace and all saliva produced was collected in a test tube during 3 min.

### 2.3 Microbial sampling

The sites sampled were the dorsum of the tongue, buccal mucosa (bilaterally), and supragingival plaque. The methods for microbial sampling have been described previously (Almståhl et al., 2017). Briefly, samples from the tongue was collected with sterile cotton pellets from a standardized area of the tongue, samples from the buccal mucosa with sterile cotton sticks, and supragingival samples from four interdental spaces with sterile tooth picks. The samples were transferred to bottles with VMGA III, which were shaken on a Whirlymixer for 10 s. Thereafter 0.1 ml was placed and stroked in a standardized fashion (Dahlén et al., 1982) to Rogosa agar plates (Neogen, Lansing, MI), which were incubated in 36°C for 5–7 days.

The microorganisms were semi‐quantified according to Dahlén et al. (1982) based on their typical colony morphology. The growth was registered as no growth, very sparse (<10 colonies), sparse (>10–100 colonies), moderate (growth also in the second stroke) and heavy (growth also in the third stroke). From their growth on Rogosa agar plates, the most dominating colony types (1–2 types) were isolated and Gram‐stained to verify that they were Gram‐Positive rods. Thereafter the isolates were re‐cultivated until purity and were then stored at −80°C. All *Lactobacillus* isolates showing moderate or heavy growth from the 21 patients (*n* = 66) were selected for identification to species level.

### 2.4 Bacterial identification

*Lactobacillus* isolates were cultured on Rogosa agar plates, which had been incubated in 90% N_2_ and 10% CO_2_ at 36°C for 3–5 days. Four of the isolates grew poorly and were excluded. For the remaining 62 isolates, bacterial colonies corresponding to approximately 1 μl were harvested. The isolates were treated with Fermentas GeneJet Genomic DNA Purification Kit (ThermoFisher Scientific, Sweden) using their protocol for Gram positive bacteria. For 15 of the 62 samples a too low level of genetic material was obtained and a more extensive DNA purification protocol was used as described by Teanpaisan and Dahlén (2006).

### 2.5 Polymerase chain reaction (PCR)

The PCR protocol used to amplify the 16S rRNA genes was based on a previous study (Teanpaisan et al., 2009). Briefly, four microliters of genetic material (sample) containing approximately 100 ng template DNA was mixed with the primers (8UA and 1492R) and HotMasterMix (5 PRIME, Germany). The samples were treated during 35 cycles of denaturation, annealing and extension followed by a final extension. The samples were then stored at −20°C.

### 2.6 Restriction fragment length polymorphism (RFLP)

The restriction enzymes Hpa II and Hae III (Thermo Scientific) were used. The genetic material from the PCR was treated according to instructions from the manufacturer. To the wells of one 5% Mini‐PROTEAN® TBE Gel (Bio‐Rad) 8 μl of samples treated with Hae III and 2 μl of sample buffer was added. To wells of another gel samples treated with HpaII and 2 μl of sample buffer was added. Nine wells were used for samples and one for a Molecular ruler (AmpliSize® Molecular Ruler, 50‐2000 base pairs, BioRad). The electrophoresis was run at 100 V for 50 min. The gel was then stained with a Silver staining kit (Plus One™, DNA Silver Staining Kit, GE Healthcare, Sweden) and dried with Drying Solution (Invitrogen, Waltham, MA).

### 2.7 *Lactobacillus* species identification

The patterns of the strains on the dried gels were compared with the patterns on gels of 13 type strains included in a previous study (Almståhl et al., 2010).

### 2.8 Acid producing capacity

*Lactobacillus* isolates were cultivated on Rogosa agar plates. After 48 h of incubation, one colony was transferred to broth (Brain Heart Infusion [BHI – Bacto™]). The tubes were incubated overnight in 90% CO_2_ and 10% N_2_, at 36 °C and harvested at the mid‐exponential phase. On the day of the experiment, the cultured bacterial cells were centrifuged for 5 min at 1300*g* and the pellet washed twice with Phosphate buffered saline (PBS) and diluted to an optical density OD_650_ nm of 1.0. A slightly modified version of the fermentation assay, described by Hedberg et al. (2008), was used to test the acid production from sugars and sugar alcohols. The methodology has been described previously (Almståhl et al., 2017). The acid‐producing ability was determined by the use of a visual 3‐graded scale as yellow (strong acid‐producing capacity, pH < 5), red/brown (weak acid‐producing capacity, pH ≥5–≤6), or purple (low/absent acid‐producing capacity, pH > 6).

In four cases, two isolates from the same time‐point and site had been collected. In three of these cases, they showed an identical acid‐producing capacity pattern and it was assumed that the isolates belonged to the same species. Only one of these isolates was therefore used in the analysis and the number of isolates was reduced from 62 to 59.

### 2.9 Anti‐microbial activity

Four oral pathogens were selected as target microorganisms: *S. mutans* (OMGS 2482), *Candida albicans* (OMGS 3750), *Staphylococcus aureus* (OMGS 3871), and *Enterococcus faecalis* (OMGS 3632). *Streptococcus mutans* is associated with caries and *C. albicans*, *S. aureus* and *E. faecalis* are associated with oral mucosal infections. All of these species are found in increased levels in irradiated patients (Almståhl et al., 2008; Almståhl et al., 2015). The method used for testing the antimicrobial activity was based on previous experiments at the Department of Oral Microbiology and Immunology (Dahlén et al., 2012). *Lactobacillus* isolates were cultivated on Rogosa agar for 48–72 h. Target microorganisms were cultivated on blood agar during 48 h at 36°C and then in Brain Heart infusion broth (BHI broth) over night. From the BHI broth with an optical density (OD)_600_ of 0.8–1.0, 0.5 ml was mixed with melted BHI‐agar and let to solidify for 20 min. Thereafter the *Lactobacillus* isolates were spread over an area of about 1.5 cm^2^. The plates were incubated for 48 h in an atmosphere appropriate for each target microorganism. Inhibition was determined visually as no inhibition (no clear zone), weak inhibition (clear zone of ≤1 mm) or strong inhibition (clear zone >1 mm). The antimicrobial activity test was performed once.

### 2.10 Statistical methods

Crosstabs with Fisher's exact test were used to analyze differences in acid‐producing capacity from sugars and sugar‐alcohols between the different *Lactobacillus species*; *L. paracasei*, *L. fermentum*, *L. casei/rhamnnosus*, others (i.e., other species [*n* = 10] plus the unidentified strain). The relationship between caries lesions/new filled surfaces at 2 years post RT (dichotomized) and presence of *Lactobacillus* with antimicrobial activity against *S. mutans* was analyzed with cross tabs and phi correlation coefficient. Regression analysis was performed to determine a possible correlation between salivary secretion rate and caries lesions/new filled surfaces. The relationship between growth of *S. mutans* at 6 and 12 months post RT, respectively, and the presence of *Lactobacillus* with antimicrobial activity against *S. mutans* at the same time‐points were also performed with cross tabs and phi correlation coefficient. *p*‐values <0.05 were considered statistically significant.

## 3 RESULTS

*Lactobacillus* isolates were collected from 21 patients, 13 men and eight women with a mean age of 59 ± 8 years (Table 1). The most common diagnoses were tonsil cancer (10 patients) followed by tongue base cancer (five patients) (Table 1). The most common treatment modality was external radiotherapy combined with chemotherapy (nine patients) followed by external radiotherapy and brachytherapy (iridium‐implant) plus chemotherapy (seven patients). The number of patients with hyposalivation (≤0.7 ml/min) was four (19%) at pretreatment, 15 (71%) at 6 months post treatment and 13 (62%) at 12 months post treatment. Seven of the patients (33%) had caries lesions, which had been restored at 24 months post treatment. For the 12 patients (9 men, 3 women) with no/very sparse/sparse growth of *Lactobacillus*, the most common diagnosis was tonsil cancer (nine patients) and the most common treatment modality external radiotherapy combined with chemotherapy (four patients). The mean salivary secretion was 2.2 ± 0.9 ml/min pretreatment, 0.7 ± 0.6 ml/min at 6 months post treatment, and 1.0 ± 0.7 ml/min and at 12 months post treatment. None had hyposalivation pretreatment, seven patients (58%) at 6 months post treatment, and five patients (42%) at 12 months post treatment. Six of the 12 patients (50%) showed no caries lesions/new filled surfaces 24 months post treatment and the other six had between 1 and 6 caries lesions/new filled surfaces. There was no correlation between salivary secretion rate and number of caries lesions/new filled surfaces at 24 months post treatment. Also, the mean secretion rate in those who had caries lesions/new filled surfaces and those with caries lesions/new filled surfaces was comparable, 0.8 ± 0.6 ml/min and 0.8 ± 0.5 ml/min.

**TABLE 1:** Age, sex (male = M, female = F), number of teeth at pretreatment, tumor site, treatment (ERT = external radiation therapy, IR = iridium implant, CYT = cytostatics, S = surgery), IMRT = intensity‐modulated radiotherapy, stimulated salivary secretion rate, and number of caries lesions/new filled surfaces at 24 months post RT for the 21 patients

Patient no	Age	Sex	No of teeth	Tumor site	Treatment	IMRT (Y/N)	Salivary secretion rate (ml/min)			No of caries lesions 24 mo post RT
							Pretreatment	6 mo post RT	12 mo post RT	
1	50	M	28	Tongue base	ERT, IR	N	3.0	1.7	1.7	0
2	51	F	28	Tonsil	CYT, ERT, IR	N	1.4	0.4	0.4	0
3	66	M	25	Tongue base	CYT, ERT, IR	Y	1.0	0.8	0.3	1
4	58	M	25	Tongue base	CYT, ERT	Y	2.4	0.7	0.9	0
5	55	F	22	Tumor colli	S, RT	N	0.8	0	0	0
6	66	F	14	Tongue base	CYT, ERT, IR	Y	1.3	0.7	0.7	2
7	56	M	24	Tonsil	CYT, ERT, IR	Y	3.0	0	0.2	1
8	55	F	30	Tumor colli	S, ERT	Y	2.8	0.5	0.8	0
9	65	M	16	Tumor colli	S, ERT	Y	0.7	0	0	0
10	63	M	24	Tonsil	CYT, ERT, IR	Y	2.5	0.8	1.6	0
11	57	M	27	Tonsil	CYT, ERT, IR	N	3.2	0.9	1.1	0
12	51	F	24	Tonsil	CYT, ERT	Y	0.7	0.1	0.6	0
13	76	M	30	Tonsil	CYT, ERT	Y	0.5	0.1	0.3	0
14	68	F	21	Tongue base	CYT, ERT, IR	Y	1.7	0	0.6	0
15	57	F	31	Tonsil	CYT, ERT	Y	2.2	0.2	0.4	0
16	65	M	25	Tonsil	ERT	Y	1.9	0.7	0.9	5
17	56	M	21	Tonsil	CYT, ERT	Y	0.7	0	0	3
18	43	M	26	Nasopharynx	CYT, ERT	Y	2.4	0.7	1.0	0
19	53	F	29	Tonsil	CYT, ERT	Y	2.5	1.5	1.4	33
20	68	M	24	Oropharyngeal	CYT, ERT	Y	2.6	0.07	0.4	9
21	60	M	25	Oropharyngeal	CYT, ERT	Y	0.9	0.1	0.2	0
Mean	59	13 M/	25			17 Y/	1.8	0.5	0.7	2.6
SD	8	8 W	4			4 N	0.9	0.5	0.5	7.3

Fifty‐eight of the 59 *Lactobacillus* isolates were identified to species level. Eight different *Lactobacillus* spp. were identified. The most common species were *L. paracasei* (*n* = 21) followed by *L. casei/rhamnosus* (*n* = 17), *L. fermentum* (*n* = 10). Other species identified were *L. plantarum* (*n* = 4), *L. delbrucheii* (*n* = 2), *L. brevis* (*n* = 2), *L. acidophilus* (*n* = 1), and *L. salivarius* (*n* = 1). Eight of the *Lactobacillus* isolates had been collected pretreatment, 24 at 6 months post‐treatment and 27 at 12 months post‐treatment.

*L. paracasei* were slightly more frequently found at 12 months post treatment compared with pretreatment and 6 months post treatment. The prevalence of *L. fermentum* was highest at 6 months post treatment (Table 2). Sixty‐seven percent of the patients harbored *L. paracasei* either at 6 or 12 months post radiotherapy. The corresponding figures for *L. casei/rhamnosus* and *L. fermentum* were 62% and 33%, respectively. At 6/12 months post treatment, *L. paracasei* was found in the supragingival plaque of nine patients and on the tongue in eight patients. *L. casei/rhamnosus* was more common in the supragingival plaque than on the tongue (10 and 3 patients), while it was the other way around for *L. fermentum* (two and five patients).

**TABLE 2:** *Lactobacillus* isolates from the tongue, supragingival plaque and buccal mucosa isolated at pretreatment, 6 and 12 months post treatment for the 21 patients

Species	Pretreatment	6 months post treatment	12 months post treatment
Tongue			
*L. paracasei*	2	4	5
*L. casei/rhamnosus*	1	3	0
*L. fermentum*	2	3	2
Other	0	1	3
Supragingival plaque			
*L. paracasei*	0	2	7
*L. casei/rhamnosus*	1	5	5
*L. fermentum*	1	2	0
Other	1	2	2
Buccal mucosa			
*L. paracasei*	0	1	0
*L. casei/rhamnosus*	0	0	2
*L. fermentum*	0	0	0
Other	0	1	1
Total			
*L. paracasei*	2	7	12
*L. casei/rhamnosus*	2	8	7
*L. fermentum*	3	5	2
Other	1	4	6

### 3.1 Acid‐producing capacity irrespective of time‐point for collection

**FIGURE 1:**
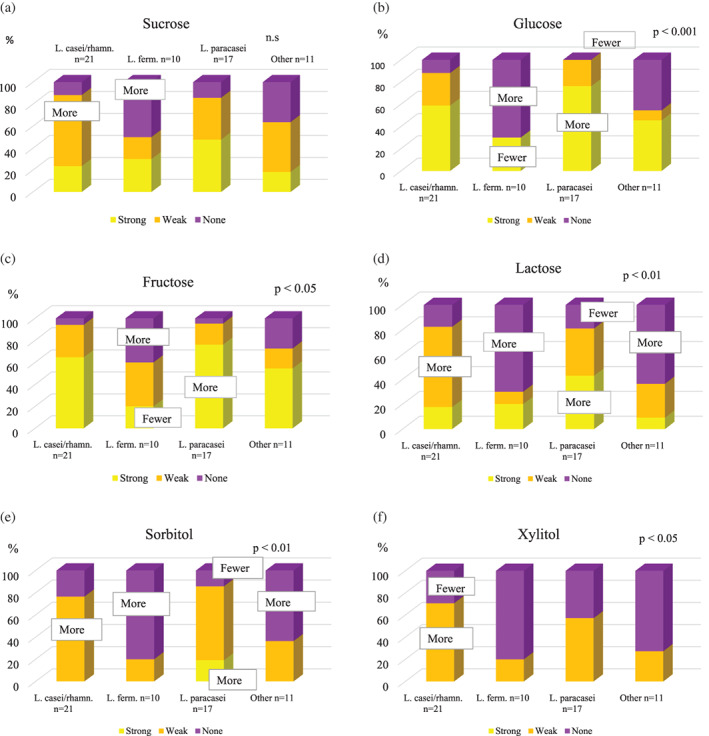
Acid‐producing capacity for the *L. casei/rhamnosus* strains (*n* = 17), *L. fermentum* strains (*n* = 10), *L. paracasei* strains (*n* = 17) and other *Lactobacillus* species (*n* = 11). Yellow = strong capacity, orange = weak capacity, and purple = no/absent capacity. Differences have been analyzed with Fisher's exact test. The *p*‐values indicate statistically significant differences in acid‐producing capacity. “Fewer” = a significantly lower number of strains compared with the other species (*p* < 0.05) and “more” = a significantly higher number compared with the other strains (*p* < 0.05) Please update the figure to the new one attached. In the Xylitol figure we have changed to More/fewer

The proportion of isolates, which showed strong acid‐producing capacity by the use of sucrose, glucose, fructose and lactose was highest among the *L. paracasei* isolates (43%–76%) (Figure 1(a–d)). There were significant differences regarding acid‐producing capacity using glucose (*p* < 0.001, Figure 1(b)), fructose (*p* < 0.05, Figure 1(c)), and lactose (*p* < 0.01, Figure 1(d)). For the sugar‐alcohols tested there were significant differences in acid‐producing capacity using both sorbitol (*p* < 0.01, Figure 1(e)) and xylitol (*p* < 0.05, Figure 1(f)). Strong acid‐producing capacity using sorbitol was only found for *L. paracasei* (19%) (Figure 1(e)). The highest proportion of strains showing weak acid‐producing capacity using xylitol was seen for *L. casei/rhamnosus* (60%) (Figure 1(f)).

### 3.2 Acid‐producing capacity for strains isolated at different time‐points

**FIGURE 2:**
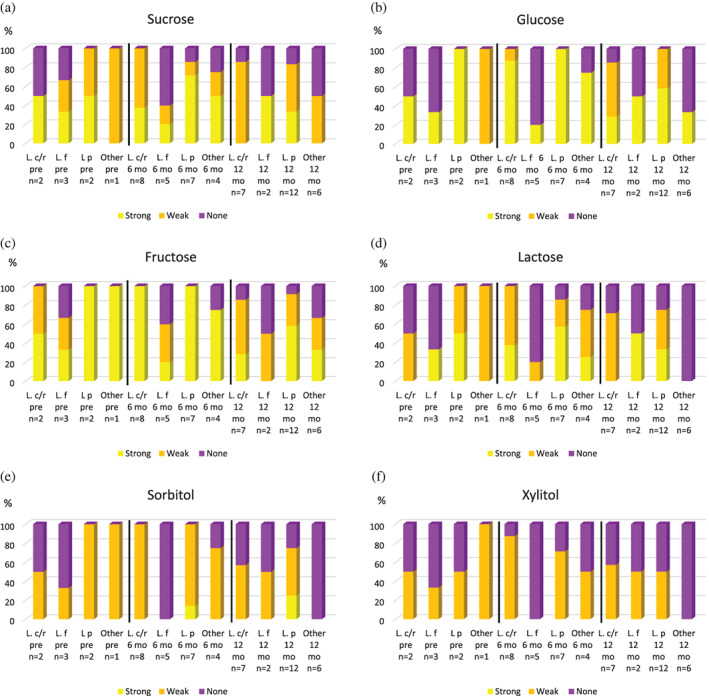
Acid‐producing capacity for *Lactobacillus* strains isolated at different time‐points (pre = pretreatment, 6 mo = 6 months post treatment, 12 mo = 12 months post treatment). L. p = *L. paracasei*, L. f = *L. fermentum*, L. c/r = *L. casei/rhamnosus*. Yellow = strong capacity, orange = weak capacity, and purple = no/absent capacity

The highest proportion of strains with strong acid‐producing capacity using both sugars (Figure 2(a–d)) and weak acid‐producing capacity using sugar‐alcohols (Figure 2(e, f)) was found among isolates collected at 6 months post treatment.

### 3.3 Acid‐producing capacity for strains isolated from the tongue and plaque

*L. casei/rhamnosus* and *L. fermentum* strains isolated from the plaque had in general higher acid‐producing capacity compared with strains isolated from the tongue. For *L. paracasei* it was the other way around for several sugars/sugar‐alcohols.

### 3.4 Anti‐microbial activity

None of the 59 isolates showed anti‐microbial activity against *C. albicans* or *E. faecalis* and only one strain against *S. aureus* (an *L. acidophilus* isolate collected from the tongue 12 months post treatment showed strong anti‐microbial activity). In Table 3, the anti‐microbial activity for the 58 isolates is shown. Forty‐two (40 = weak) isolates (72%) showed an anti‐microbial activity against *S. mutans*. The highest proportion of isolates with anti‐microbial activity against *S. mutans* was seen for *L. paracasei* (81%) followed by *L. fermentum* (70%) and *L. casei/rhamnosus* (56%). There was no correlation between growth of *S. mutans* in the supragingival plaque and presence of *Lactobacillus* with anti‐microbial activity either at 6 months (phi: 0.09, *p* = 0.68) or 12 months post radiotherapy (phi: −0.05, *p* = 0.79). Both patients with no growth and patients with heavy growth of *S. mutans* harbored *Lactobacillus* with anti‐microbial activity against *S. mutans*. Also, there was no correlation between caries lesions/new filled surfaces at 2 years post radiotherapy and presence of *Lactobacillus* with anti‐microbial activity against *S. mutans* post radiotherapy (phi: −0.09), *p* = 0.52). The highest proportion of isolates with anti‐microbial activity was seen at pretreatment, 88% followed by 6 months post treatment 71% (Table 3).

**TABLE 3:** Inhibition of *S. mutans* for the 58 *Lactobacillus* isolates

Species	Time‐point					
	Pretreatment		6 months post treatment		12 months post treatment	
	Yes	No	Yes	No	Yes	No
*L. paracasei* (*n* = 21)	2	0	6	1	9	3
*L. casei/rhamnosus* (*n* = 17)	1	1	6	2	3	4
*L. fermentum* (*n* = 10)	3	0	2	3	2	0
*L. plantarum* (*n* = 4)	1	0	1	0	1	1
*L. brevis* (*n* = 2)	—	—	1	1	—	—
*L. delbruckeii* (*n* = 2)	—	—	—	—	1	1
*L. acidophilus* (*n* = 1)	—	—	—	—	1	0
*L. salivarius* (*n* = 1)	—	—	—	—	1	0
Unknown (*n* = 1)	—	—	1	0	—	—
Sum	7	1	17	7	18	9

## 4 DISCUSSION

To the best of our knowledge, there are no previous reports on the prevalence of oral *Lactobacillus* species and their acid‐producing capacity and anti‐microbial activity at different time‐points in relation to treatment of cancer of the head and neck region. The most prevalent species among the 21 patients were *L. paracasei*, *L casei/rhamnosus*, and *L. fermentum* of which *L. paracasei* was the most prevalent species at 12 months post treatment. The highest proportion of isolates showing strong acid‐producing capacity using sugars was found for *L. paracasei* of which four also showed strong acid‐producing capacity using sorbitol. The highest proportion showing weak acid‐production capacity using xylitol was seen for *L. casei/rhamnosus* both at 6 and 12 months post treatment. Anti‐microbial activity was seen for one isolate against *S. aureus* and for 42 isolates (72%) against *S. mutans*. All but one patient had *Lactobacillus* with antimicrobial activity against *S. mutans* at least at one time‐point. Antimicrobial activity was most prevalent among *L. paracasei* (81%).

In our previous study, using the same method for identification, the most frequently isolated species from plaque in irradiated patients 3–5 years post treatment were *L. fermentum* 29%, *L. casei* 22%, *L. rhamnosus* 19%, and *L. paracasei* 7% (Almståhl et al., 2010). In the present study, a slightly lower prevalence of *L. casei/rhamnosus* was found in the supragingival plaque, 33% while the prevalence of *L. paracasei* was much higher, 53%. There was also a difference in the prevalence of *L. fermentum* since no *L. fermentum* was isolated from the plaque at 12 months post radiotherapy in the present study. A possible explanation to the discrepancies might be that in the former study several isolates were collected from either anterior or posterior tooth surfaces (Almståhl et al., 2010), while in the present study only 1–2 isolates was collected from samples where plaque from four sites had been pooled. It is also possible that the microbial composition changes over time after treatment of cancer of the head and neck.

### 4.1 Acid‐producing capacity and species

In accordance with our previous study (Almståhl et al., 2010), the proportion of *L. fermentum* isolates showing strong acid‐producing capacity using sugars and sugar‐alcohols was lower compared with *L. paracasei* and *L. casei/rhamnosus*. A high proportion of the *L. paracasei* isolates was able to decrease the pH to <5.0 using sucrose, glucose and fructose, which is also in accordance with the results of our previous study (Almståhl et al., 2010). It has been shown that it takes a longer time for the pH to reach 5.5 using glucose for *L. fermentum*, 4.18 h, compared with *L. casei/paracasei*, 2.87 h, and *L. rhamnosus* 2.27 h (Piwat et al., 2012). Also, the final pH is higher for *L. fermentum*, pH 4.58, compared with *L. casei/paracasei*, pH 4.02 and *L. rhamnosus* pH 3.89 (Piwat et al., 2012). In our previous study, the proportion of strains able to produce acids using sorbitol was highest among *L. paracasei* (49%), while *L. fermentum* rarely had that ability (Almståhl et al., 2010). These results are also in line with those in the present study where 19% of the *L. paracasei* isolates showed a strong acid‐producing capacity using sorbitol and 66% weak; while only 20% of the *L. fermentum* isolates had this ability (Figure 2(b)).

### 4.2 Anti‐microbial activity

In the present study, anti‐microbial activity was determined on agar plates where growth inhibition of the test microorganism was registered. This method has been used for the determination of the anti‐microbial activity of enterococcus strains at out laboratory (Dahlén et al., 2012). *Lactobacillus* spp. can have several activities or abilities, which can contribute to inhibition of other species such as bacteriocins, bi‐products of fermentation such as lactic acid and hydrogen peroxide, or their ability to reduce the pH (Slover & Danziger, 2008). With the method used in the present study, no information about which activities or abilities that were responsible for the anti‐microbial effect can be obtained. Also, only one strain of *S. mutans*, *S. aureus*, *C. albicans*, and *E. faecalis* was tested. It was surprising that only one *Lactobacillus* isolate showed anti‐microbial activity against *S. aureus* and none against *C. albicans* or *E. faecalis*. This indicates that the marked increase in *Lactobacillus* seen in most patients post radiotherapy might not contribute to the defense against mucosal pathogens. Further studies are needed to examine the anti‐microbial activity of *Lactobacillus* isolates and to elucidate the mechanisms involved.

All but one patient had *Lactobacillus* with anti‐microbial activity against *S. mutans* at least at one time‐point. High proportions of *Lactobacillus* with anti‐microbial activity against *S. mutans* have been reported also in other studies (Köll et al., 2008; Köll‐Klais et al., 2005; Simark‐Mattson et al., 2009). Contrary to our hypothesis, the highest proportion of strains with antimicrobial activity was found pretreatment. The number of isolates collected pretreatment was however low making it difficult to explain these results. Most isolates showed week anti‐microbial activity, thus a possible inhibitory effect on *S. mutans* in vivo is questionable.

*Lactobacillus* spp. from patients without caries experience have been shown to have a better inhibitory effect on *S. mutans* than *Lactobacillus* from patients with arrested or active caries (Simark‐Mattson et al., 2007). In the present study, we found no correlation between presence of *Lactobacillus* spp. with anti‐microbial activity against *S. mutans*, and growth of *S. mutans*, and no correlation with presence of caries lesions/new filled surfaces at 2 years post radiotherapy. This lack of correlation may be due to the relatively low number of patients included in the present study. Other plausible explanations may be that also other bacteria are involved in caries development like for example *Scardovia wiggsiae* and *Bifidobacterium dentium* (Henne et al., 2015; Zhou et al., 2016). In addition, other factors are involved in the caries process such as the amount and quality of saliva, intake frequency of easily fermentable carbohydrates, oral hygiene level, and fluoride exposure (Fejerskov et al., 2015).

In the present study, the highest proportion of isolates with anti‐microbial activity against *S. mutans* was also found among *L. paracasei*, 81%, which is in line with previous results (Simark‐Mattson et al., 2009). However, a high proportion of *L. fermentum* also showed anti‐microbial activity, 70%, while the one *L. fermentum* included in the study by Simark‐Mattson et al. (2009) could not inhibit *S. mutans*. A plausible explanation for the divergent results is that different populations were included, Simark‐Mattson et al. (2009) isolated *Lactobacillus* from children and young adults, while in the present study *Lactobacillus* were isolated from patients with a mean age of 59 years who had undergone treatment of cancer of the head and neck region.

### 4.3 Methodological considerations

With the method used for identification, *L. casei* and *L. rhamnosus* could not be distinguished since their patterns on the gel was identical. To differentiate between these two species, they could have been subjected to sodium dodecyl sulphate‐polyacrylamide gel electrophoresis (SDS‐PAGE) for whole cell protein analysis (Almståhl et al., 2010; Teanpaisan & Dahlén, 2006). However, little is known about differences between these two species and it is unlikely that such information would have added much to the results. With the method used for species identification it was not possible to determine whether the same strain was isolated at different time‐points or if it were other strains. To include methods identifying strains to further analyze variations in prevalence of *Lactobacillus* and their characteristics might give further knowledge about *Lactobacillus* and their role in the oral cavity. Only *Lactobacillus* from head and neck cancer patients were included on not from healthy controls. *Lactobacillus* are not part of the oral microbiome at good oral health (Bik et al., 2010). Also, we included healthy controls with normal salivary secretion rate in our previous study (Almståhl et al., 2013), but only a few had detectable *Lactobacillus*.

## 5 CONCLUSIONS

The most frequent species isolated from head and neck cancer patients both pretreatment and post treatment were *L. paracasei*, *L. casei/rhamnosus*, and *L. fermentum* of which *L. paracasei* showed the best acid‐producing capacity and the highest proportion with anti‐microbial activity against *S. mutans*.

## CONFLICT OF INTEREST

The authors have no conflict of interest.

## Data Availability

The data that support the findings of this study are available from the corresponding author upon reasonable request.
